# hucMSC Conditioned Medium Ameliorate Lipopolysaccharide-Induced Acute Lung Injury by Suppressing Oxidative Stress and Inflammation via Nrf2/NF-*κ*B Signaling Pathway

**DOI:** 10.1155/2021/6653681

**Published:** 2021-08-13

**Authors:** Yue Tang, Fengxia Ding, Chun Wu, Bo Liu

**Affiliations:** ^1^Department of Cardiothoracic Surgery; Ministry of Education Key Laboratory of Child Development and Disorders; National Clinical Research Center for Child Health and Disorders; China International Science and Technology Cooperation Base of Child Development and Critical Disorders, Children's Hospital of Chongqing Medical University, Chongqing, China; ^2^Chongqing Key Laboratory of Pediatrics; Chongqing Engineering Research Center of Stem Cell Therapy, Chongqing Medical University, Chongqing, China; ^3^Department of Respiratory Medicine; Ministry of Education Key Laboratory of Child Development and Disorders; National Clinical Research Center for Child Health and Disorders; China International Science and Technology Cooperation Base of Child Development and Critical Disorders, Children's Hospital of Chongqing Medical University, Chongqing, China

## Abstract

Acute lung injury (ALI) is a common clinical syndrome in the cardiac intensive care unit with a high mortality rate. Inflammation and oxidative stress have been reported to play a crucial role in the development of ALI. Previous studies have shown that human umbilical cord mesenchymal stem cells (hucMSCs) have anti-inflammatory and antioxidative effects in various diseases. However, the anti-inflammatory and antioxidative effects of the hucMSC conditioned medium (CM) on LPS-induced ALI remain unclear. Therefore, in this study, we assessed whether the hucMSC conditioned medium could attenuate LPS-induced ALI and the underlying mechanisms. Mice were randomly divided into four groups: the control group, PBS group, LPS+PBS group, and LPS+CM group. The lung histopathology and bronchoalveolar lavage fluid (BALF) were analyzed after intervention. The Nrf2/NF-*κ*B signaling pathway and its downstream target genes were tested, and the cytokines and growth factors in CM were also measured. The results showed that CM significantly attenuated the histological alterations; decreased the wet/dry weight ratio; reduced the levels of MPO, MDA and ROS; increased SOD and GSH activity; and downregulated the level of proinflammatory cytokines such as IL-1*β*, IL-6, and TNF-*α*. Furthermore, CM promoted the expression of Nrf2 and its target genes NQ01, HO-1, and GCLC and inhibited the expression of NF-*κ*B and its target genes IL-6, IL-1*β*, and TNF-*α*. These effects may be closely related to the large amounts of cytokines and growth factors in the CM. In conclusion, our results demonstrated that CM could attenuate LPS-induced ALI, probably due to inhibition of inflammation and oxidative stress via the Nrf2/NF-*κ*B signaling pathway.

## 1. Introduction

Acute lung injury (ALI) is a frequent cause of acute respiratory failure with high morbidity along with mortality [1]. It is mainly characterized by the alveolar epithelium and endothelium injuries, neutrophil and macrophage infiltration, and pulmonary vascular permeability increase, which led to alveolar-capillary barrier disruption, pulmonary edema, and impaired gas exchange [2]. Up to now, the mechanisms of ALI have not been well elucidated, and there are still neither effective measures nor specific drugs to treat this disease. Research evidence opines that inflammation coupled with oxidative stress play an indispensable role in the onset and progression of ALI [3, 4]. After ALI occurs, the lung tissue develops a strong inflammatory reaction and the inflammatory cells infiltrate into the alveolar space to result in the generation of proinflammatory inflammatory cytokines along with chemokines consisting of TNF-*α* (tumor necrosis factor-alpha), IL-1*β* (interleukin-1*β*), and IL-6 (interleukin-6). At the same time, the aberrant release of ROS (reactive oxygen species) could further aggravate the process of lung injury [4].

NF-*κ*B constitutes a pivotal inflammatory transcription cytokine involved in inflammation and immune response, and upon activation, it causes the release of various proinflammatory cytokines. Nrf2, a key transcription cytokine of antioxidant responses, could alleviate oxidative stress damage by modulating various cytoprotective enzymes. Recent investigations have shown that there is a close relationship of the NF-*κ*B with the Nrf2 signaling cascades. Nrf2 activators have been found to alleviate NF-*κ*B signaling through dampening the phosphorylation of IKK/I*κ*B along with the nuclear translocation of the p65 NF-*κ*B subunit [5]. In contrast, NF-*κ*B directly represses the activation of the Nrf2 signaling cascade at the transcriptional level. NF-*κ*B and Nrf2 compete for the gene transcription activator CBP (CREB-binding protein). Furthermore, NF-*κ*B recruits histone deacetylase 3 (HDAC3), causing local hypoacetylation to antagonize the Nrf2 pathway [6]. Therefore, the synergistic targeting of the NF-*κ*B and Nrf2 pathways may be an important therapeutic approach for ALI.

Lipopolysaccharide (LPS), the primary constituent of the outer membrane of Gram-negative bacteria, can cause a series of inflammatory reactions and oxidative stress responses. LPS induces excessive secretion of proinflammatory cytokines, ROS along with chemokines, thus making it an ideal substance for inducing ALI [2]. Human umbilical cord mesenchymal stem cells (hucMSCs) have been used to treat numerous diseases such as pulmonary fibrosis, nephropathy, and myocardial ischemic injury [7]. Previous investigations documented that hucMSCs have anti-inflammatory, antioxidative stress, and antifibrotic influences on LPS-triggered ALI [8]. However, the effect of hucMSC conditioned medium (CM) on LPS-triggered ALI remains unclear. Herein, we explored the therapeutic effect of the hucMSC conditioned medium on LPS-triggered ALI in mice and its responsible mechanism.

## 2. Materials and Methods

### 2.1. Ethics Statements

The research work involving human along with animals was conducted, per the principles of the Helsinki Declaration, and was granted approval by the Ethical Committee of Chongqing Medical University (File No. 2018-055). All animal experimental producers were reviewed, as well as approved by the Animal Care and Use Committee of Children's Hospital of Chongqing Medical University.

### 2.2. Preparation of hucMSCs Conditioned Medium

hucMSCs were isolated, cultured, and identified according to our previous methods [9]. The third passage hucMSCs were cultured in a T75 flask to 80-90% confluence. We rinsed the cells thrice with PBS and introduced serum-free DMEM (10 ml) to the culture flask, and incubation was continued for 48 hours. The medium was collected and span at 1500 rpm for 5 minutes to get rid of the cell debris. CM was concentrated 25-folds by using centrifugal filter units with a centrifugal filter device (3 kDa cut-off, Millipore, USA) and then filtered with a 0.22 *μ*m syringe filter (Corning). CM was maintained at -80°C until use.

### 2.3. Animals and Treatment Protocols

Male C57BL/6 mice (8-12 weeks), weighing 20-25 g, were commercially obtained from the Experimental Animal Center of Chongqing Medical University. We maintained the study mice under standard laboratory settings at the Laboratory Animal Center for Children's Hospital of Chongqing Medical University with provision of standard food and water. An ALI model was established as previously documented [10]. Concisely, we anesthetized the mice with sodium pentobarbital and then intratracheally administered with LPS (5 mg/kg in 50 *μ*l PBS, E. coli 055: B5, Sigma-Aldrich, USA). After 4 hours, 200 *μ*l CM or phosphate-buffered saline (PBS) was injected via the tail vein. Twelve hours after the tail vein injection, we collected lung tissue along with the bronchoalveolar lavage fluid for further study. Mice were stratified into 4 groups (10 per group) at random: the control group, which was maintained under standard laboratory settings with standard food and water; the PBS group: normal mice were inoculated with 200 *μ*l PBS via the tail vein; the LPS+PBS group: mice were administered an intratracheal inoculation of LPS and then received 200 *μ*l PBS via the tail vein; and the LPS+CM group: mice were administered an intratracheal inoculation of LPS and then received 200 *μ*l CM via the tail vein.

### 2.4. Lung Wet to Dry (W/D) Weight Ratio

The W/D ratio was calculated to evaluate lung edema. The lungs were harvested and immediately weighed. Then, the dry weight was obtained after the lung was incubated at 80°C for 48 h.

### 2.5. Collection of Bronchoalveolar Lavage Fluid (BALF) and Inflammatory Cell Counting

We lavaged the lungs thrice using 0.5 ml of cold saline (overall volume 1.5 ml) through tracheostomy with a tracheal cannula to collect BALF. Immediately, we span the collected BALF at 1000 r/min for 10 minutes and obtained pelleted cells. Afterwards, the cells were resuspended in PBS, followed by counting of them with a hemocytometer. Wright-Giemsa staining was adopted to differentially count cells (neutrophils and macrophages).

### 2.6. Protein Concentration Assay in BALF

We lavaged the lungs thrice using 0.5 ml of cold saline (overall volume 1.5 ml) through tracheostomy with a tracheal cannula to obtain BALF. After centrifugation, a small portion of the supernatant was assayed for total protein concentration with the BCA (bicinchoninic acid) method approach.

### 2.7. Quantification of Cytokines in BALF Supernatants

The contents of IL-1*β* and IL-6 along with TNF-*α* in BALF supernatants were quantified using ELISA as described by the manufacturer (Jiancheng Bioengineering Institute, Nanjing, China).

### 2.8. Measure ROS Contents in BALF

The level of ROS contents in BALF was with test kits, per manufacturer's manuals (Jiancheng Bioengineering Institute, Nanjing, China).

### 2.9. Measurement of GSH, SOD, MDA, and MPO Contents in Lung Tissues

To analyze the GSH, SOD, MDA, and MPO contents, 12 hours following LPS inoculation, we excised the right lungs and homogenized the tissues followed by dispersion in the extraction buffer. The contents of GSH, SOD, MDA, and MPO were analyzed, per protocols of the manufacturers (Jiancheng Bioengineering Institute, Nanjing, China).

### 2.10. Histopathological Evaluation of Lung Tissues

The lower lobes of the left lung tissues were fixed in 4% formalin for 24 hours and then paraffin embedded. Afterwards, H&E staining of sections was done. Each slide was examined by two separate investigators in a blinded approach. The lung injury score was calculated as previously described [11].

### 2.11. Immunohistochemistry and Immunofluorescent

After the slices were deparaffinized and rehydrated, the antigen was retrieved by citrate buffer. 3% H_2_O_2_ was employed to block endogenous peroxidase for 10 minutes, followed by blocking of the nonspecific protein docking site with 0.5% BSA for another 1 hour. After that, we inoculated the slices overnight with 1 : 200 diluted phosphorylated NF-*κ*B p65 antibodies (Cell signaling Tech, United States) and Nrf2 antibodies (Cell signaling Tech, United States), respectively, at 4°C. After rinsing thrice with PBS, the matching secondary antibody was introduced and incubated for 1 hour. Images were observed under a microscope after nuclear counterstaining.

### 2.12. RT-PCR

The TRIzol reagent was employed to purify total RNA from lung tissues (Invitrogen, CA, USA) as described by the manufacturer. After that, cDNA was generated using the PrimeScript™ RT reagent kit. Subsequently, qPCR was done using indicated forward and reverse primers as follows:

IL-6 5′-CCCAATTTCCAATGCTCTCC-3′ (forward), 5′ -CGCACTAGGTTTGCCGAGTA′ (reverse); IL-1*β* 5′-TGCCACCTTTTGACAGTGATG-3′ (forward), 5′-TGATGTGCTGCTGCGAGATT-3′ (reverse); TNF-*α* 5′-GATCGGTCCCCAAAGGGATG-3′ (forward), 5′-TTTGCTACGACGTGGGCTAC-3′ (reverse); HO-1 5′-CGCCTTCCTGCTCAACATT-3′ (forward), 5′-TGTGTTCCTCTGTCAGCATCAC-3′ (reverse); NQ01 5′-TTCTGTGGCTTCCAGGTCTT-3′ (forward), 5′-TCCAGACGTTTCTTCCATCC-3′ (reverse); GCLC 5′-TTCCAAGCCTGCAGCATATC-3′ (forward), 5′-CAGACTCGTTGGCATCATCC-3′ (reverse); and *β*-actin 5′-CCGTAAAGACCTCTATGCCAAC-3′ (forward), 5′-GGGTGTAAAACGCAGCTCAGTA-3′ (reverse).

*β*-Actin served as an inner reference.

### 2.13. Western Blot Analysis

Total proteins from the lung tissue homogenates were extracted according to the instructions provided in the BCA protein assay kit (Beyotime, China). Fractionation of 40 *μ*g of protein samples was done with the SDS-polyacrylamide gel and transfer-embedded onto PVDF membranes. Afterwards, blocking of membranes was done with the 5% skimmed milk for one hour and inoculated overnight with primary antibodies against NF-*κ*B p65 antibodies (1 : 1000, Cell signaling Technology, United States), phosphorylated NF-*κ*B p65 antibodies (1 : 1000, Cell signaling Technology, United States), Nrf2 antibodies (1 : 1000, Cell signaling Technology, United States), and Histone H3 (1 : 1000, Cell signaling Technology, United States) in TBST (Tris-buffered saline/Tween) at 4°C. Then, we rinsed the membranes thrice in TBST and inoculated for two hours with secondary antibodies at room temperature. Immobilon Western Chemiluminescent HRP Substrate (Millipore, United States) was adopted to detect positive immune reaction.

### 2.14. ELISA

The levels of cytokines and growth factors in the hucMSC conditioned medium were assayed using ELISA kits (VEGF, HGF, NGF, and KGF, Jiamay Bitotech; IL-6, IL-8, and TGF-*β*1, Neobioscience) following the manufacturer's protocol.

### 2.15. Statistical Analyses

All data were given as mean ± standard deviation (SD). *P* < 0.05 denoted statistical significance. The mean value of each group was compared with one-way analysis of variance (ANOVA) and then with Dunnett's post hoc test. All analyses were done in SPSS software version 16.0.

## 3. Results

### 3.1. hucMSC Conditioned Medium Reduces Pulmonary Edema

**Figure 1:**
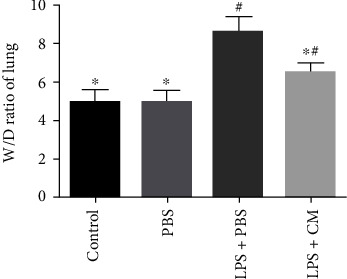
Effects of CM on W/D ratio lung tissues. CM or PBS was injected 4 h after LPS administration. The lungs were excised at 12 h after LPS challenge, and the lung W/D ratio was determined. Values presented as mean ± SD. *n* = 10 mice per group. ^∗^*P* < 0.05 versus the LPS+PBS group; ^#^*P* < 0.05 versus the control and PBS groups.

The severity of pulmonary edema was quantified via the lung W/D weight ratio. As shown in Figure 1, the lung W/D weight ratio was remarkably increased in the LPS+PBS group in contrast with the control group and the PBS group. Nonetheless, the LPS+CM group exhibited a remarkable reduction in the lung W/D weight ratio (*P* = 0.24).

### 3.2. hucMSC Conditioned Medium Attenuates the Pathological Damage of Lung Tissue

**Figure 2:**
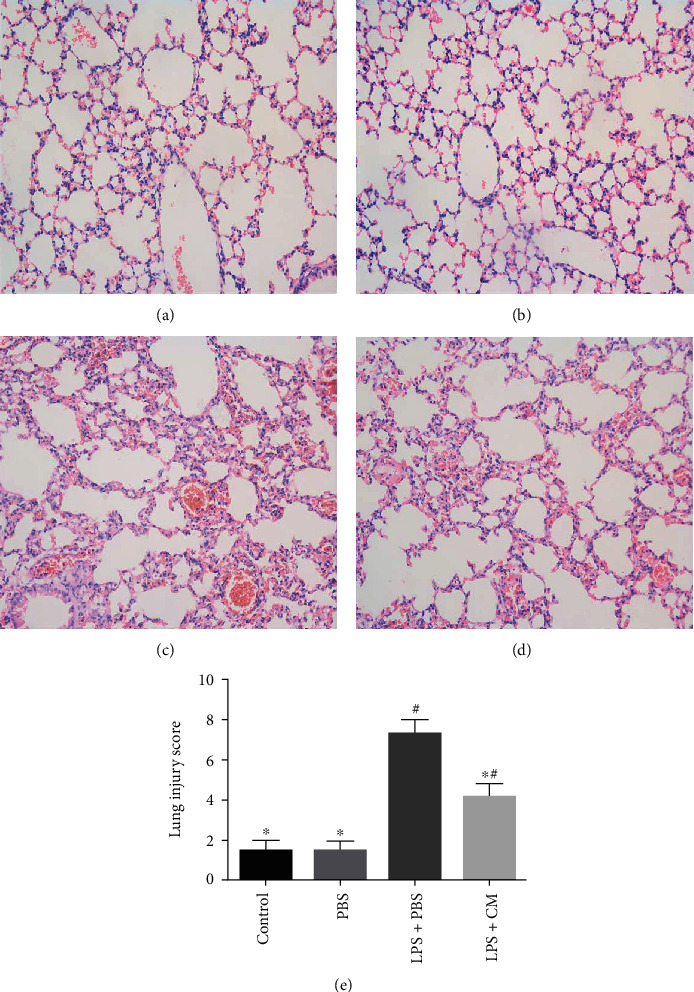
Effects of CM on LPS-induced lung histopathological changes. CM or PBS was injected 4 h after LPS administration. Lung tissues were collected at 12 h after LPS challenge for histological evaluation. (a) The control group. (b) The PBS group. (c) The LPS+PBS group. (d) The LPS+CM group. (e) Severity scores of lung injury. Values presented as mean ± SD. *n* = 10 mice per group. ^∗^*P* < 0.05 versus the LPS+PBS group; ^#^*P* < 0.05 versus the control and PBS groups.

As shown in Figure 2, the lungs in the control and PBS groups showed normal tissue structure with little or no inflammatory cell infiltration. Nevertheless, in the LPS+PBS group, alveolar hemorrhage, perivascular or interstitial edema, and thickening of the alveolar wall, along with invasion of inflammatory cells around the alveoli and capillaries were evident, and these histopathological changes were remarkably improved in the LPS+CM group. The results of lung injury score were consistent with pathological changes.

### 3.3. hucMSC Conditioned Medium Improves the Permeability and Inflammatory Response of Lung Tissue

**Figure 3:**
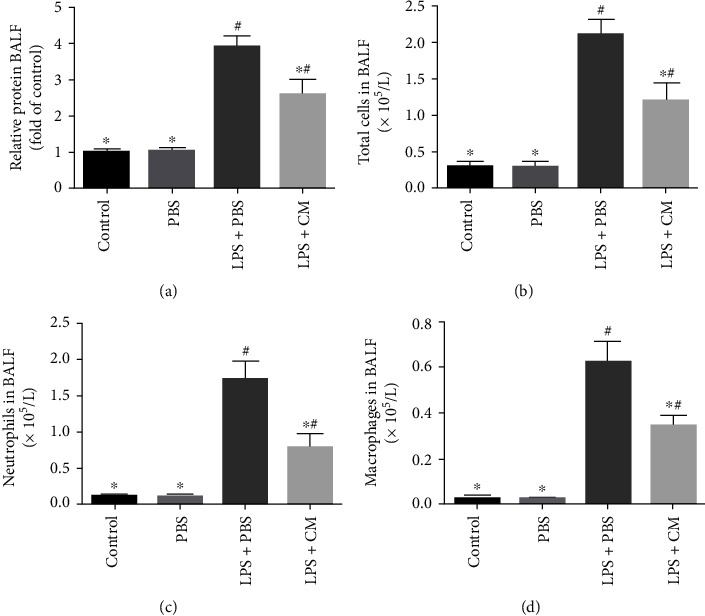
Effects of CM on LPS-induced protein leakage and inflammatory cell infiltration in BALF. CM or PBS was injected 4 h after LPS administration. BALF were collected at 12 h after LPS challenge for protein concentration assay and inflammatory cell counting. (a) Effects of CM on LPS-induced protein leakage in BALF. (b) The numbers of total cells in BALF. (c) The numbers of neutrophils in BALF. (d) The numbers of macrophages in BALF. Values presented as mean ± SD. *n* = 10 mice per group. ^∗^*P* < 0.05 versus the LPS+PBS group; ^#^*P* < 0.05 versus the control and PBS groups.

**Figure 4:**
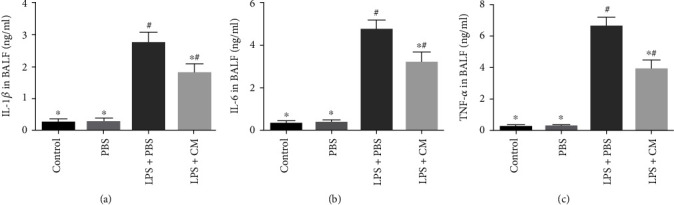
Effects of CM on LPS-induced proinflammatory cytokine secretion in BALF. BALF supernatant was collected to detect the level of proinflammatory cytokines by ELISA. (a) IL-1*β* in BALF. (b) IL-6 in BALF. (c) TNF-*α* in BALF. Values presented as mean ± SD. *n* = 10 mice per group. ^∗^*P* < 0.05 versus the LPS+PBS group; ^#^*P* < 0.05 versus the control and PBS groups.

The concentration of protein in the BALF reflects the permeability of the lung tissue. The data illustrated that the concentration of protein in the LPS+PBS group was remarkably increased, and the value was decreased after treatment with CM (*P* = 0.31). Furthermore, to assess the influence of CM on LPS-induced lung inflammation, the overall number of inflammatory cells and the number of diverse inflammatory cells in BALF were counted. Our study showed that LPS remarkably escalated the total number of inflammatory cells (2.33 ± 0.34 × 10^5^/l) as well as the number of neutrophils (1.78 ± 0.36 × 10^5^/l) and macrophages (0.63 ± 0.04 × 10^5^/l) in BALF. Interestingly, CM remarkably diminished the number of inflammatory cells as well as neutrophil and macrophage infiltration (Figure 3). Next, inflammatory cytokines in the BALF were explored by ELISA. LPS remarkably increased the levels of IL-1*β* (2.78 ± 0.41 ng/ml), IL-6 (4.42 ± 0.73 ng/ml), and TNF-*α* (6.78 ± 0.83 ng/ml), while CM remarkably reduced the production of these cytokines (Figure 4).

### 3.4. hucMSC Conditioned Medium Improves Enzyme Activity along with Oxidative Stress Damage in Lung Tissue

**Figure 5:**
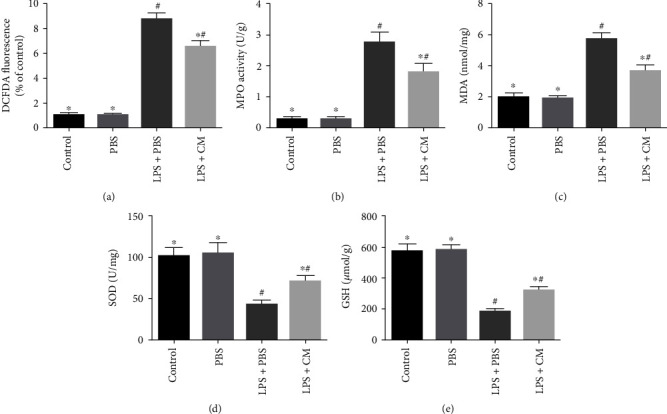
Effects of CM on LPS-induced oxidative stress products and enzyme activity in BALF and lung tissues. BALF and lung tissues were collected at 12 h after LPS challenge. (a) ROS in BALF. (b) MPO in lung tissues. (c) MDA in lung tissues. (d) SOD in lung tissues. (e) GSH in lung tissues. Values presented as mean ± SD. *n* = 10 mice per group. ^∗^*P* < 0.05 versus the LPS+PBS group; ^#^*P* < 0.05 versus the control and PBS groups.

Our study found that CM remarkably inhibited the levels of LPS-triggered MPO (*P* = 0.21). Meanwhile, we hypothesize that the possible potential protective properties of CM against LPS-triggered ALI by improving oxidant stress. We found that CM remarkably reduced ROS generation and MDA formation while remarkably increasing the contents of GSH along with SOD (Figure 5).

### 3.5. hucMSC Conditioned Medium Regulates Nrf2/NF-*κ*B Signaling Cascade to Attenuate ALI

**Figure 6:**
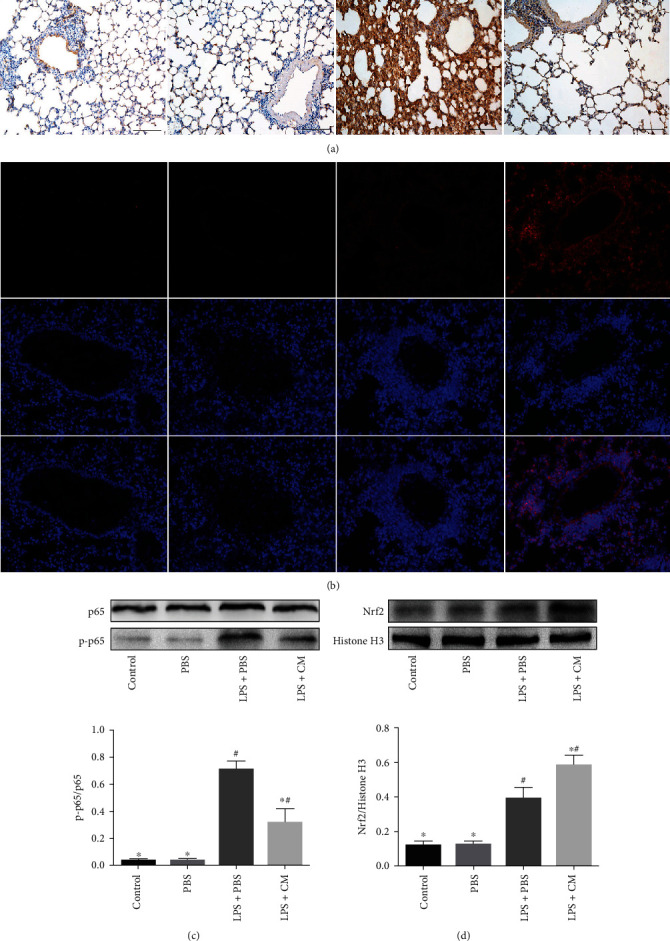
Effects of CM on LPS-induced Nrf2/NF-*κ*B signaling pathway changes in lung tissues. Lung tissues were collected at 12 h after LPS challenge for immunostaining and western blot. (a) Immunohistochemistry of phosphorylated NF-*κ*B p65. (b) Immunofluorescence of Nrf2. (c) Western blot analysis of p65 and p-p65. (d) Western blot analysis of Nrf2. Values presented as mean ± SD. *n* = 10 mice per group. ^∗^*P* < 0.05 versus the LPS+PBS group; ^#^*P* < 0.05 versus the control and PBS groups.

The Nrf2/NF-*κ*B signaling cascade plays an indispensable role in the inflammation and oxidative stress in LPS-triggered ALI. The activity of Nrf2along with NF-*κ*B was analyzed by immunostaining and western blot. As illustrated in Figure 6, phosphorylated NF-*κ*B p65 and nuclear Nrf2 were remarkably increased in lung tissue after LPS stimulation. However, CM remarkably reduced phosphorylated NF-*κ*B p65 and increased nuclear Nrf2 levels (*P* < 0.05).

**Figure 7:**
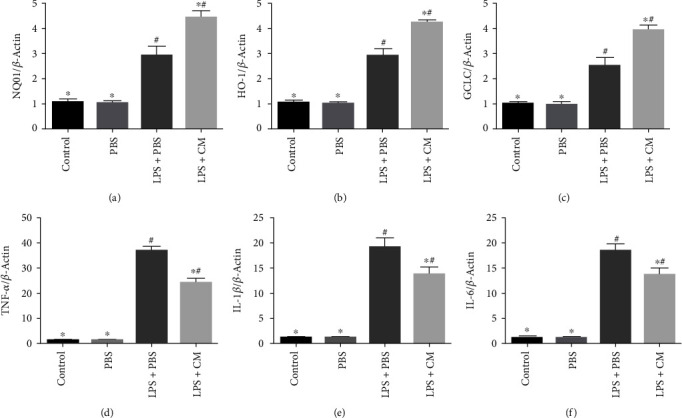
Effects of CM on target genes of the Nrf2/NF-*κ*B signaling pathway in lung tissues. Lung tissues were collected at 12 h after LPS challenge for real-time quantitative PCR analysis of (a) NQ01, (b) HO-1, (c) GCLC, (d) TNF-*α*, (e) IL-1*β*, and (f) IL-6. Values presented as mean ± SD. *n* = 10 mice per group. ^∗^*P* < 0.05 versus the LPS+PBS group; ^#^*P* < 0.05 versus the control and PBS groups.

Herein, the expression of Nrf2 target genes NQ01, HO-1 along with GCLC, and NF-*κ*B target genes IL-1*β*, IL-6, as well as TNF-*α*, was measured by PCR. As illustrated in Figure 7, LPS induced the expressions of Nrf2 and NF-*κ*B target genes, while CM increased the expression of Nrf2 target genes and inhibited NF-*κ*B target gene expression (*P* < 0.05). These results indicated that CM may simultaneously inhibit the NF-*κ*B signaling cascade and activate the Nrf2 signaling cascade in ALI.

### 3.6. Cytokines and Growth Factors in hucMSC Conditioned Medium

The levels of cytokines and growth factors in the hucMSC conditioned medium were measured using ELISA kits. The contents of VEGF, HGF, NGF, KGF, IL-6, IL-8, and TGF-*β*1 in CM were shown Table 1.

## 4. Discussion

ALI, especially acute respiratory distress syndrome, is a frequent cause of acute respiratory failure. Excessive oxidative stress and overwhelmed inflammatory response are prominent pathophysiology of ALI [4]. Previous studies found that mesenchymal stem cells could effectively alleviate ALI and protect lung function in animal models [8]. However, the clinical application of mesenchymal stem cells still faces immune rejection and ethical issues. Therefore, it is necessary to find new treatment methods to avoid these shortcomings. Herein, we explored if the anti-inflammatory and antioxidant activities of CM play a protective role in LPS-triggered ALI.

Pulmonary edema is a characteristic pathological change in ALI. The lung W/D weight ratio can be used to measure the severity of pulmonary edema [10]. We found that CM can decrease the lung W/D weight ratio and effectively reduce pulmonary edema. Further, CM can alleviate histopathological changes caused by LPS including inflammatory cell invasion, alveolar hemorrhage, alveolar collapse, interstitial edema, and alveolar wall thickening. In addition, the protein concentration, total number of cells, neutrophil count, and macrophage count in the BALF were remarkably reduced. These findings suggest that CM can effectively alleviate LPS-induced ALI.

Lung inflammation after ALI is characterized by inflammatory cell invasion, release of a large number of proinflammatory cytokines, alveolar interstitial edema, reduced lung compliance, and ultimately impaired lung function [4]. In the early phases of ALI, neutrophils are one of the first immune cells to produce an immune response and migrate to the site of inflammation. MPO is an enzyme mainly present in neutrophil azurophilic granules, and its activity reflects the number and activity of neutrophil infiltration and thus is an important index for evaluating the severity of inflammation [12]. Our study found that the MPO activity was remarkably increased after LPS exposure, while the MPO activity was remarkably repressed by CM. LPS stimulates monocytes and macrophages to produce endogenous cytokine TNF-*α*, which can induce an inflammatory cascade and aggravate lung injury [13]. IL-6 is also a pivotal cytokine involved in LPS-triggered ALI and plays an indispensable role in inflammatory and cellular immunity [14]. IL-1*β* synergizes with TNF-*α* along with IL-6 to induce chemokine production and participate in the inflammatory cascade [15]. We established that CM remarkably diminished the contents of TNF-*α*, IL-1*β*, and IL-6, suggesting that the protective influence of CM is due in part to repression of the inflammatory response.

LPS exposure can not only cause the secretion of inflammatory cytokines but also produce a large amount of ROS, aggravating inflammation and tissue damage. Excessive production of ROS is one of the pathogenesis of ALI [16]. Our study found that the ROS production was remarkably increased by LPS and was remarkably inhibited by CM. MDA is a lipid peroxidation product that can be utilized as a marker of oxidative stress [17]. We found that LPS can remarkably increase MDA formation, while CM can inhibit its formation. Further, we tested the activities of antioxidant enzymes consisting of GSH and SOD. The data illustrated that CM remarkably dampened the reduction of GSH and SOD in lung tissue caused by LPS.

To further clarify the underlying mechanism by which CM reduces inflammation and oxidative stress in ALI, we explored the influence of CM on the Nrf2/NF-*κ*B signaling cascade. Previous reports have documented that Nrf2 and NF-*κ*B are key molecules for LPS-induced oxidative stress and inflammatory responses, respectively [18, 19]. Nrf2 is modulated by Keap1 and modulates the expression of antioxidant proteins, as well as phase II detoxification enzymes by interacting with antioxidant response element (ARE). During oxidative stress occurs, Nrf2 dissociates from Keap1, and activated Nrf2 enters the nucleus, inducing expression of downstream target genes such as NQ01, HO-1, and GCLC. Accumulating evidences indicated that NF-*κ*B has an indispensable role in managing the release of proinflammatory cytokines consisting of TNF-*α*, IL-1*β*, and IL-6. Recent investigations have documented the Nrf2 cascade, and the NF-*κ*B cascade is mutually inhibited at their transcriptional level by protein-protein cross-talks or by affecting the second messenger [5, 6]. Therefore, we explored the influence of CM on the Nrf2/NF-*κ*B signaling cascade. We found that CM can promote the expression of Nrf2 and its target genes NQ01, HO-1, and GCLC and inhibit the expression of NF-*κ*B and its target genes TNF-*α*, IL-1*β*, and IL-6. These results demonstrate that CM can reverse the imbalance of LPS-induced Nrf2/NF-*κ*B signaling pathways.

Finally, we found that CM contains a variety of cytokines and growth factors, which may be a potential protection mechanism of ALI. VEGF induces endothelial cell migration by inducing the expression of matrix metalloproteinases and promotes the formation of a new capillary network. In addition, VEGF can also inhibit the inflammatory response by inhibiting leukocyte adhesion [20]. HGF has a regulatory effect on various tissues and cells and has a close relationship with cell growth, differentiation, and angiogenesis [21]. NGF can provide nutrition for neurons and promote neurite outgrowth. It also regulates immunity in the nonneurological system, reduces the production of proinflammatory cytokines, escalates the release of anti-inflammatory cytokines, and promotes the repair of tissue damage [22]. KGF can directly induce the proliferation of bronchial and alveolar epithelial cells and promote the repair of damaged epithelium [23]. Similarly, IL-6, IL-8, and TGF-*β*1 are involved in immune regulation and tissue damage repair [24].

There are several limitations of the present study. We used commercial LPS to induce ALI, while LPS-induced inflammation cannot fully recapitulate bacterial infections in humans. Secondly, although CM has been observed to have a good therapeutic effect in mouse models of ALI, we still need to prove the effectiveness of CM treatment in humans with ALI. Thirdly, CM has a complicated composition, and recent studies have shown that exosomes secreted by stem cells into CM are also closely related to damaged tissue repair [25]. Therefore, the involvement of stem cells in injury repair is a comprehensive result, and its mechanism needs further study.

## 5. Conclusions

In conclusion, our study shows that hucMSC CM has a protective influence on LPS-triggered ALI. CM can reduce inflammation along with oxidative stress damage triggered by LPS. The mechanism by which CM protects against lung injury may be closely related to maintain the balance of the Nrf2/NF-*κ*B signaling cascade. CM may be a prospective treatment for ALI, but the underlying mechanisms need further investigation before clinical application.

## Figures and Tables

**Table 1:** Cytokines and growth factors in hucMSC conditioned medium. The conditioned medium was collected after incubating with serum-free DMEM for 48 hours. Levels of VEGF, HGF, NGF, KGF, IL-6, IL-8, and TGF-*β*1 were measured by using ELISA.

Cytokines and growth factors	Assay	CM (*n* = 3, pg/ml)
VEGF	ELISA	490.58 ± 62.35
HGF	ELISA	1794.36 ± 89.73
NGF	ELISA	57.41 ± 8.24
KGF	ELISA	34.58 ± 5.04
IL-6	ELISA	1012.40 ± 320.52
IL-8	ELISA	942.40 ± 401.72
TGF-*β*1	ELISA	2046.40 ± 102.73

## Data Availability

Please contact author for data requests.
